# Distinct Soil Bacterial Communities Revealed under a Diversely Managed Agroecosystem

**DOI:** 10.1371/journal.pone.0040338

**Published:** 2012-07-23

**Authors:** Raymon S. Shange, Ramble O. Ankumah, Abasiofiok M. Ibekwe, Robert Zabawa, Scot E. Dowd

**Affiliations:** 1 Department of Agricultural and Environmental Science, Tuskegee University, Tuskegee, Alabama, United States of America; 2 United States Department of Agriculture-Agricultural Research Service-United States Salinity Lab, Riverside, California, United States of America; 3 George Washington Carver Agricultural Experiment Station, Tuskegee University Tuskegee, Alabama, United States of America; 4 Molecular Research LP, Shallowater, Texas, United States of America; University of Delaware, United States of America

## Abstract

Land-use change and management practices are normally enacted to manipulate environments to improve conditions that relate to production, remediation, and accommodation. However, their effect on the soil microbial community and their subsequent influence on soil function is still difficult to quantify. Recent applications of molecular techniques to soil biology, especially the use of 16S rRNA, are helping to bridge this gap. In this study, the influence of three land-use systems within a demonstration farm were evaluated with a view to further understand how these practices may impact observed soil bacterial communities. Replicate soil samples collected from the three land-use systems (grazed pine forest, cultivated crop, and grazed pasture) on a single soil type. High throughput 16S rRNA gene pyrosequencing was used to generate sequence datasets. The different land use systems showed distinction in the structure of their bacterial communities with respect to the differences detected in cluster analysis as well as diversity indices. Specific taxa, particularly Actinobacteria, Acidobacteria, and classes of Proteobacteria, showed significant shifts across the land-use strata. Families belonging to these taxa broke with notions of copio- and oligotrphy at the class level, as many of the less abundant groups of families of Actinobacteria showed a propensity for soil environments with reduced carbon/nutrient availability. Orders Actinomycetales and Solirubrobacterales showed their highest abundance in the heavily disturbed cultivated system despite the lowest soil organic carbon (SOC) values across the site. Selected soil properties ([SOC], total nitrogen [TN], soil texture, phosphodiesterase [PD], alkaline phosphatase [APA], acid phosphatase [ACP] activity, and pH) also differed significantly across land-use regimes, with SOM, PD, and pH showing variation consistent with shifts in community structure and composition. These results suggest that use of pyrosequencing along with traditional analysis of soil physiochemical properties may provide insight into the ecology of descending taxonomic groups in bacterial communities.

## Introduction

Land use change and management in the conversion of forested, pastured, and cropped land have ecosystem-scale impacts such as: soil cycling of organic compounds [1]–[3], biodiversity [4]–[5], and soil nutrient dynamics [6]. The sensitivity of microbial communities to changes in management as well as their importance to nutrient cycling is a reason why they have been considered as early indicators of change in the quality of the soil ecosystem [7]. Although changes in bacterial communities have been reported in prior research [8], [9], advances in sequencing technology have provided researchers with the ability to assess bacterial diversity at lower costs, and quicker turnaround than prior 16S rRNA and sequencing methods. These advances have allowed researchers to enhance work in the much needed area of bacterial community structure at varying scales [10]–[12].

Physical disturbance of the soil has been reported as being a crucial factor in determining soil biotic characteristics in agroecosystems [13]. The loss/disturbance of a stratified soil microhabitat has been attributed to a decrease in the density of species that inhabit agroecosystems. It has been proposed that such soil biodiversity reductions may be negative because the recycling of nutrients and proper balance among organic matter, soil organisms and plant diversity are necessary components of a productive and ecologically balanced soil environment [14]. For example, Acosta-Martinez et al. [15] evaluated the physical disturbance to bacterial communities with respect to tillage and reported that tillage reduced the bacterial diversity due to the interruption to physical diversity of the soil environment. Torsvik et al. [16] conducted a study comparing biodiversity of bacterial communities in perturbed soils versus relatively undisturbed, pristine soils, and found that human-induced pollution can lead to profound changes in microbial community structure by greatly reducing bacterial diversity. Other studies that have introduced organic amendments to soils have showed changes in microbial community structure in addition to biochemical changes [17], [18].

**Table 1:** Sequences Recovered, Observed and Predicted OTUs for each sample.

Sample	Total Sequences Recovered	OTUs	ACE	Chao1
Cultivated 1	10,860	2065	7709	4699
Cultivated 2	5,318	589	1326	1009
Cultivated 3	11,494	1272	3150	2253
Forested 1	11,812	990	3094	2088
Forested 2	15,724	914	2104	1626
Forested 3	6,402	359	743	596
Pastured 1	12,382	1470	5491	3461
Pastured 2	19,981	1488	5174	3461
Pastured 3	18,678	1767	6816	4171

16S rRNA gene sequences recovered from soil DNA for each sample and the corresponding OTUs identified for each richness estimate.

Three primary areas of land use in the southeastern United States include pine plantation, annual cropping, and livestock pastures. The incorporation of forestry (particularly pine plantation) into agroecosystems has become an important economic, social [19], and environmental [20] issue in ecosystem management of the southeastern USA. Grazing of pine forests to control understory growth is one of the management systems which have been proposed to further diversify agroecosystems, and manage fire hazards [21], [22]. All three of these land use types have the potential to alter soil chemical and physical disturbance, and ultimately to influence changes in soil bacterial community structure and composition. The ecological impact of management practices has been demonstrated as being a consistent source of disturbance to soil ecosystems [23]. Participating landowners and farmers are often engaged in multiple management practices within the same agroecosystem, creating a level of ecological complexity that is difficult to replicate in controlled settings. Hobbs and Huenneke [23] concluded that such disturbance has the potential to alter plant communities, but this process is poorly understood in the case of microbial species. It is therefore the objective of this study to utilize 16S rRNA sequencing techniques to elucidate the bacterial community structure and composition that exists in a single soil type exhibiting the three land use types discussed above. DNA was extracted from soils under each type of land use introduced above, amplified, and subjected to pyrosequencing. We therefore hypothesized that the specified land use types across the landscape will exhibit distinct soil bacterial communities.

## Results and Discussion

### Richness and Diversity Estimates

**Figure 1:**
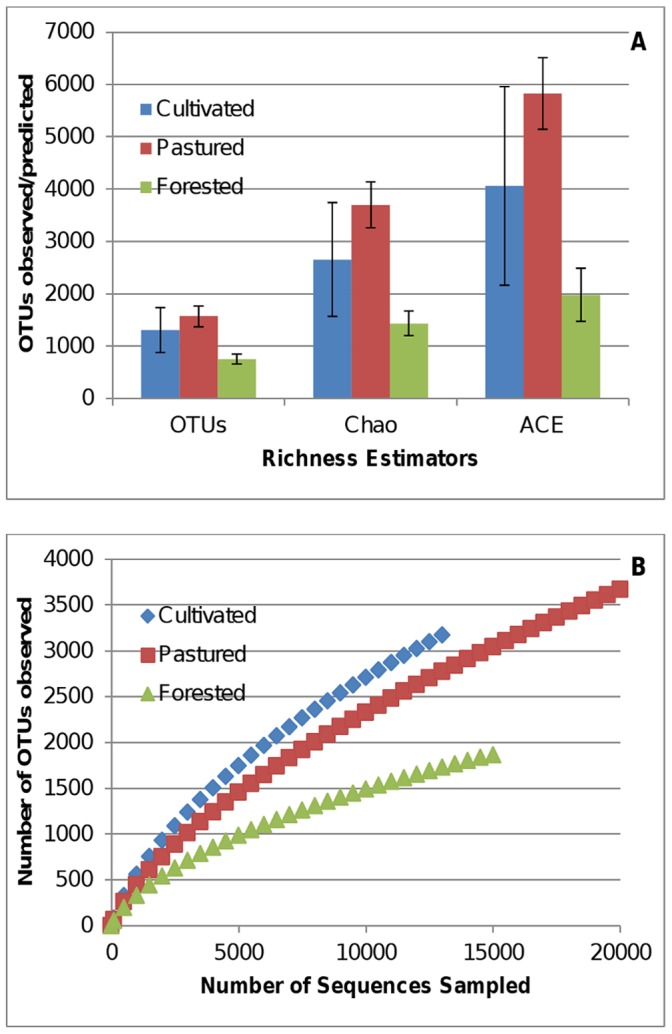
Diversity estimates. Richness/diversity estimators (**a**) and rarefaction curves (**b**) are presented as calculated by MOTHUR at a level of 3% dissimilarity.

The maximum operational taxonomic units (OTUs) detected across the study site according to the observed clusters (sobs) at 3% dissimilarity was 2065 (Table 1) in the Cultivated sample 1, despite there being an obvious dominance of sequence recovery from the pastured samples. The maximum amount of OTUs observed is also reflected in the Chao and ACE values predicted for that sample. Though the highest number of OTUs was observed and predicted for this particular sample, the richness values were quite variable. All four of the estimators followed a distinct trend (pine forested<cultivated<grazed pasture) (Figures 1a and 1b). That trend exemplified an agroecosystem in which the lowest number of OTUs was found in soils sampled under the forest land use system and the highest under the grazed pasture soils. The indices reflected the same trend when calculated at 5% dissimilarity; however no differences were found between the land management systems for any of the diversity estimators.

In the richness/diversity data presented in Figures 1a and 1b, the grazed pasture system further establishes this point as its richness trends higher than the pine forest for all three of the estimators and trended higher than the community under the cultivated system. Increased richness in semi-stable pastures can be expected, but the added effects of grazing has been reported by other researchers as serving to enhance microbial communities attributing to three major factors: organism and substrate diversification from fecal and urine deposition, stimulation rhizosphere activity as a result of mowing, and the mixing and dispersal of microbial communities through trampling [24]–[26]. This pattern is not only noticeable in richness, but is also reflected in the highest SOC, TN, PD, and APA values (Table 2). Though the richness values of the cultivated community are not as high as those of the grazed pasture, they do not show a significant difference. In prior studies [27], [28] no significant differences were detected between agricultural and pastured soils. It may be assumed that bacterial community richness under cultivated management would differ due to habitat difference introduced by inorganic amendments and tillage, but Acosta-Martinez et al. [11] suggest diverse crop residues and root systems may be cause for increased richness in diversely planted cultivated soils.

**Table 2:** Selected soil properties.

*Soil Properties*	Cultivated	Forested	Pastured
pH (H_2_O)	6.08a	4.90b	6.06a
SOC (g kg^−1^ soil)	2.70a	3.69b	4.41c
TN (g kg^−1^ soil)	0.26a	0.28a	0.39b
*APA* †	1.43a	2.10b	3.21b
*ACP* †	3.60a	3.30a	3.73a
*PD* †	1.62a	1.10b	1.96a
Sand‡	0.17a	0.31b	0.32b
Silt‡	0.34a	0.30b	0.27b
Clay‡	0.49a	0.39b	0.40ab

Means of selected soil properties and their means amongst different land use strata. Different letters denote significant differences between stratified land uses at P≤0.05 (n = 45).

† Values for enzyme activity are in units of µmol p-nitrophenol g soil^−1^ hr^−1^.

‡ Values for particle size are expressed as a fraction of total soil particles (1.00).

APA = acid phosphatase ACP = alkaline phosphatase PD = phosphodiesterase.

SOC = soil organic carbon TN = total nitrogen.

The forested system is the most distinguishable land use regime according to the acidic pH (Table 2) and the reduced amount of OTUs detected in the communities. In figures 1a & 1b, the forested soil bacterial community consistently shows the lowest values for richness. The forested soil presents harsh conditions to resident organisms, specifically in the acidic pH and polyphenolic compounds found in loblolly pine litter [29]. Soil pH has been found in numerous studies that utilize 16S sequencing techniques, as the driving abiotic factor shaping community structure in soils [30], [9], [12], [31]. Though not directly measured in the study, polyphenolic compounds resulting from leaf litter have been reported as having a negative impact on soil bacterial communities [32], [33] as well. This chemical disturbance creates a community with distinct abiotic and biotic characteristics, which impacts the growth and stability of the soil bacterial community.

### Relative Abundance of Bacterial Phyla and Classes

**Figure 2:**
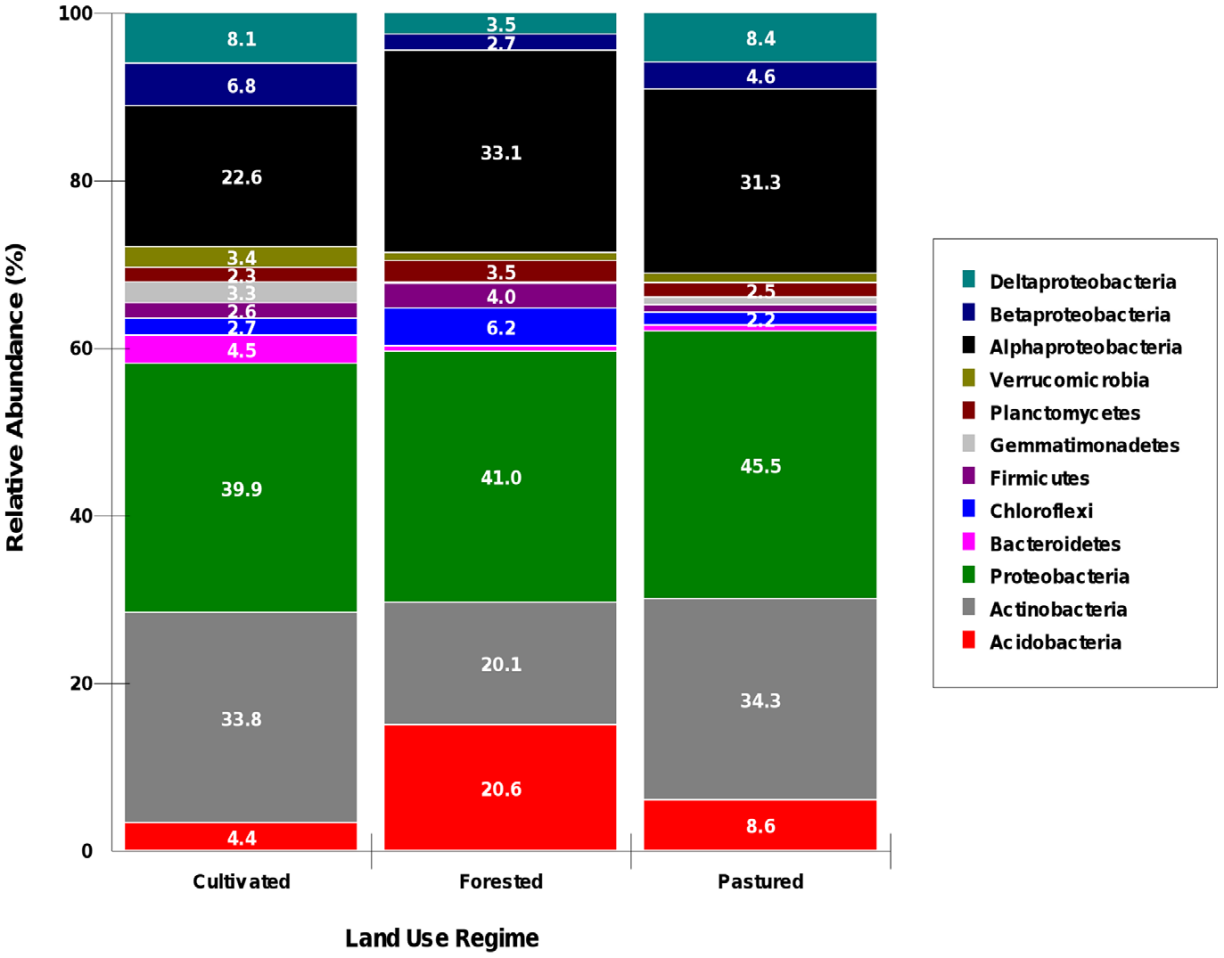
Relative abundance of major taxonomic groups across land use systems. Phyla included in this figure had relative abundance values consistently greater than 1%, as well as the abundant classes of the phylum Proteobacteria. Values presented are the mean percent. CLT = Cultivated; GRP = Grazed Pasture; PNF = Pine Plantation.

Bacterial community compositions of the soils were examined at descending levels of biological classification to determine the effect of diversified land use on community membership. Detailed phylogenetic analyses grouped the soil associated bacterial sequences into 26 phyla (including unknown). The relative abundances of the 10 most abundant phyla are presented in Figure 2. The phylum distribution showed that Proteobacteria was the most dominant phyla with mean relative abundance values ranging from 39.9 % in cultivated soil, to 41.0 % in forested soil and to 45.5% in pastured soil. Other dominant phyla of note were Actinobacteria (representing 20.1–34.3% of the bacterial sequences in the samples) and Acidobacteria (which represented 4.4–20.6% of the bacterial sequences in each sample). Results from running GLM substantiate that the land uses are significantly different when considering the composition of the bacterial communities. Specific differences were observed between the cultivated and forested land use types for the phyla Acidobacteria (P<0.01), while Actinobacteria showed significant distinction (P<0.05) under the pine forested land use system.

The remaining phyla accounted for fewer than 25% of the relative abundance observed and were designated as minor (reference to abundance only). Of these groups, Gemmatimonadetes showed significantly distinguishable populations in all three land use types (forested/cultivated, P<0.001; forested/pastured, P≤0.001; cultivated/pastured, P<0.05), while Chloroflexi (forested/cultivated, P<0.05; forested/pastured, P<0.05) relative abundance is distinct in the forested system, and Verrucomicrobia (pastured/cultivated, P<0.05; forested/cultivated, P<0.05), BRC1 (pastured/cultivated, P<0.001; forested/cultivated, P<0.001) and Nitrospirae (pastured/cultivated, P<0.001; forested/cultivated, P<0.001) showed significant distinction in the cultivated area. Another significant difference observed the pastured and cultivated land use was in the phyla Cyanobacteria (P≤0.005). Shannon-Wiener indices for evenness calculated at 3% dissimilarity showed that the cultivated system had the highest value of evenness (0.93) compared to the pastured (0.89) and forested (0.87) with significant differences (P<0.05) occurring between the cultivated and forested systems. When considering the difference of the composition of the bacterial community under the cultivated land use compared to the other management systems, it has been suggested [34] and supported [35] that the cultivation of a field, no matter how long ago, leaves an indelible imprint on the soil bacterial community. Through tillage, organic matter is incorporated throughout the plough layer of the soil and benefits unique microbial communities as the community reverts to an earlier (and more unstable) stage of ecological succession [36]. These communities are described as having quick responses to conditions of feast or famine that would be created by growing season-fallow pattern in cultivated systems. Figure 2 exhibits this phenomenon as the minor phyla in the cultivated system accounted for almost 20% of the sequences detected, and showed a more even distribution of abundance. With the values of evenness presented above, the data provides further evidence of a more even community in the cultivated system disturbed by tillage.

At the class level, α-proteobacteria, Acidobacteria (class), and Actinobacteria (class) were the dominant bacterial classes found across the site. Of the classes of Proteobacteria, α-proteobacteria appeared to be the most dominant among them. The remaining classes of Proteobacteria (with exception to γ-proteobacteria) showed higher relative abundance compared to that of any other taxa at the class level (excluding class Actinobacteria). Based on GLM of transformed data, relative abundance was significantly higher for α-proteobacteria and Acidobacteria in the forested than the cultivated system, while β-proteobacteria and δ-proteobacteria were significantly higher in the cultivated system (N = 3; P<0.05). Seemingly, the pastured area was transitionary in its bacterial class abundance, as it differs only from the forested system only including an increase in Acidobacteria (N = 3; P<0.01) and a decrease in β-proteobacteria and δ-proteobacteria (N = 3; P<0.05). The observed shifts in taxonomic groups may suggest that microbial community composition changes in response to land management or environmental perturbation.

It has recently been reported that Actinobacteria and β-proteobacteria follow copiotrophic lifestyles [37], [38], while Acidobacteria can be classified as oligotrophic. The results of the other soil properties present the land use regimes as environments that could support both types of lifestyles. The copiotrophic environment could be considered as the pastured system which has significantly higher SOC, TN, and nutrient cycling due to the activity of alkaline phosphatase and phosphodiesterase. The oligotrophic environment could be then considered as the forested system which is lacking in SOM and enzymatic activity as compared to the pastured system. The trend with respect to the two trophic groups can be seen in Figure 2, in which the class Actinobacteria and the classes of Proteobacteria (with the exception of α-proteobacteria) trend down in the forested system with the opposite being observed for Acidobacteria. The instability of the cultivated community may also explain the lowest values for key taxa in the study. Even though α-proteobacteria was not explicitly designated as an oligotroph, in this study, as well as in other studies [27], [31], its members have shown the ability to outcompete most (if not all other) bacterial classes in acidic soils. What this data suggests is that the chemical ecology of the forested system may provide the foundation for significant differences in community membership as well as growth. Studies have also shown that shifts from forest to grassland soil [31] as well as cultivated to pasture [11] result in changes to bacterial community composition. The assertion of trophic lifestyles was stated as a general guideline, so a deeper look into descending taxonomic groups was observed for these particular classes to determine if this assertion holds at these levels.

**Figure 3:**
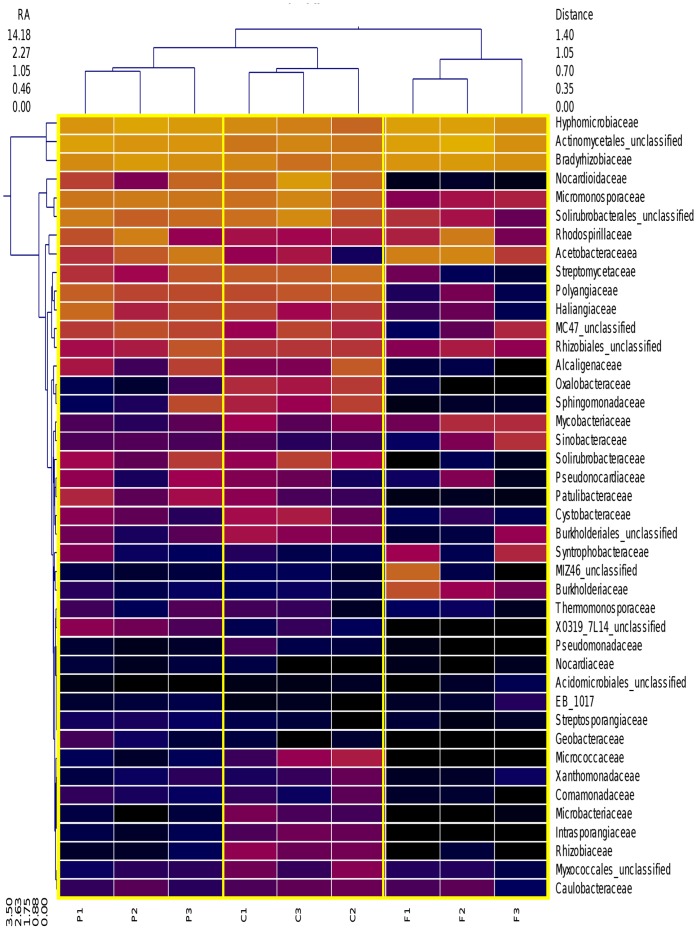
Hierarchical cluster analysis presented as a double dendrogram. A double cluster dendrogram that demonstrates the relative abundance of Families across the 9 samples across the three land use systems. Clustering in the Y-direction is indicative of abundance, not phylogenetic similarity. RA = Relative Abundance; CLT = Cultivated; PST = Grazed Pasture; FST = Grazed Pine Plantation.

A heatmap (with hierarchal clustering) (Figure 3) of the bacterial families found in the samples was generated with respect to the classes previously mentioned as following copiotrophic/oligotrophic lifestyles. The heatmap demonstrates that the top three families found in the study belong to the most abundant classes Actinobacteria and α-proteobacteria. The forested system appears to have the most distinction and was verified with ANOVA. The forested system differed from the other two systems significantly (P<0.05; n = 3) for the families: Acidobacteriaceae, Solibactereaceae, Solirubrobacterales (unclassified), Solirubrobactereaceae, Patullibactereaceae, Micromonosporaceae, Streptomycetaceae, Nocardioidaceae, Sphingomonodaceae, Alcaligenaceae, Burholderiaceae, Haliangiaceae, Polyangiaceae, Pseudomonadaceae. Out of the 42 Families graphed from these groups, less than half show abundance patterns that would support this assertion. As these are generally the most abundant families, prior assertions may be biased to those groups who are most identifiable using current methods. The evenness of the bacterial community under the cultivated system that was observed in Figure 2 and the reported Shannon evenness values is again exemplified by abundance spread further across the specified families than in any of the other systems. This is shown by the coverage of purple to orange colors in the columns labeled for the cultivated samples. The opposite is true for the Forested system, in which there is a large and visible gap in class abundance (exhibited by the large amount of black cells) in the columns designated as those belonging to the forested system. The samples of the Pastured and Cultivated systems seem to be more closely related in composition, as their clustering in the X-direction shows divergence at ∼1.05, while the Forested system diverges at ∼1.40 from the other two. Wardle et al. have also suggested that microbial community diversity/richness may not respond to cultivation in such a way that can distinguish it from a community with fewer disturbances [39].

### Diversity and Abundance of Actinomycetes

To further demonstrate the differences in bacterial community composition, relative abundance was assessed at the level of Order for Actinobacteria as well. Table 3 displays the relative abundance and Shannon diversity indices of the most salient orders of Actinobacteria identified in the soils across the three land use types. The orders Solirubrobacterales, Actinomycetales, 0319-7L14, MC47, Acidomicrobiales, and Unclassified Actinobacteria (class) were identified as contributing substantially to the relative abundance of this class. Generally, both relative abundance and the Shannon index were lowest in the forested system for all Orders of Actinobacteria with the exception of Acidomicrobiales, which actually had the highest relative abundance and was second to the pastured system with respect to diversity.

**Table 3:** Abundance and diversity of orders from class actinobacteria.

Orders of Actinobacteria	Cultivated		Forested		Pastured	
	RA^a^	SI^b^	RA^a^	SI^b^	RA^a^	SI^b^
*Solirubrobacterales*	6.21	3.15	1.67	2.23	2.93	4.00
*MC47*	1.86	2.52	1.03	2.38	2.37	2.88
*0319-7L14*	0.39	1.12	0	N/A	0.98	2.07
*Acidomicrobiales*	0.05	1.40	0.34	1.56	0.15	1.69
*Actinomycetales*	24.04	4.97	16.88	3.65	23.36	3.83
*Unclassified*	0.27	2.04	0.22	0.97	0.31	1.92

The mean relative abundance and Shannon index values as calculated for the Orders of the Class Actinobacteria.

a Relative abundance (%) of taxonomic group with respect to total OTUs observed for community.

b Shannon diversity index.

These results suggest that pH shapes the Acidobacterial community structure more than the other environmental variables measured. Although the chemical environment seems to play a major role in the patterns of abundance, some of the taxa seem to respond to other factors. From the orders of Actinobacteria detected across the study site it is observed that Actinomycetes are the most abundant order (Table 3), and show a trend in their relative abundance and diversity across the site that reflects the amount of disturbance (pine forested<grazed pasture≤cultivated). Actinomycetes have been shown to have a preference for animal and human activity [40], and may be a possible biomarker in determining effects of land use change. Prior studies utilizing different primers from various geographic locations have suggested that Actinomycetes show higher abundance in agricultural and pasture soils, [41], [42], [9]. Although Solirubrobacterales has not been extensively studied, recent studies have shown its members to be adaptive in their ability to colonize different ecosystems: fungal growing ant colonies [43], spinach phyllosphere [44], desert and Antarctic soil [45], [46]. In this study, the order seems to favor physical disturbance as its highest relative abundance is in the cultivated system, and its highest diversity is in the pastured system. In this way, Solirubrobacterales performs similar to Actinomycetales with respect to disturbance.

### Principle Coordinates Analysis

**Figure 4:**
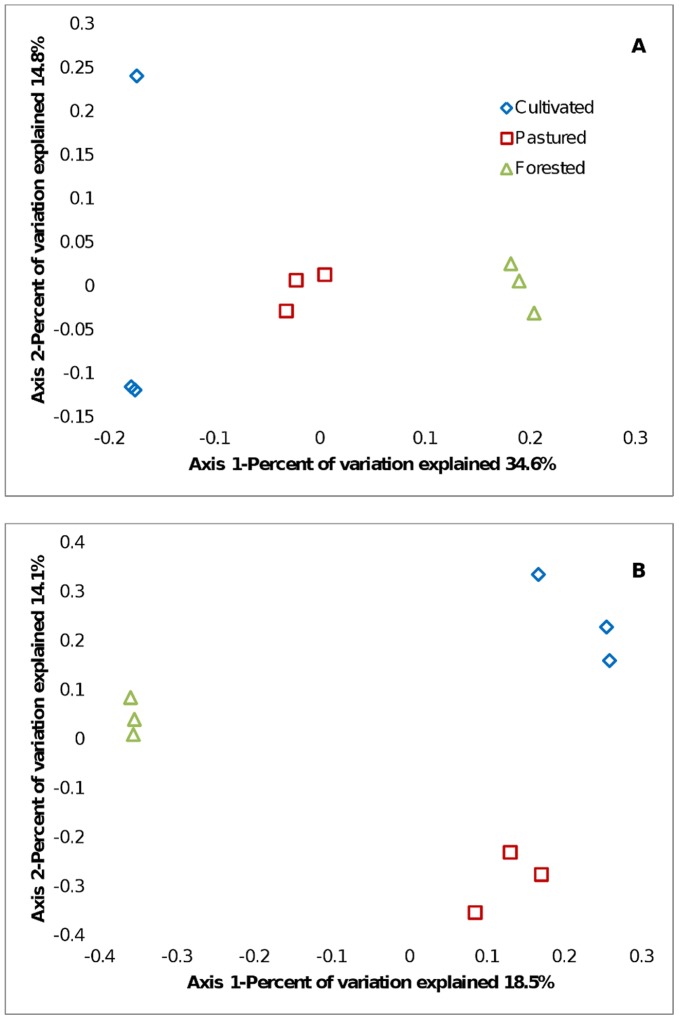
Ordination of unifrac metrics. PCoA plots are presented of the first two axes based on (a) weighted and (b) unweighted Unifrac distance matrices showing the quantitative and qualitative clustering of samples. The pastured system is represented by the red; the cultivated by the yellow; and the forested by the green.

Unifrac metrics were used to assess community similarity between two or more samples according to their structure (weighted/quantitative) and membership (unweighted/qualitative). In the 2-dimensional plot visualized from the Unifrac weighted distance matrix principle coordinates analysis (3% dissimilarity), the samples of each system distinctively responded to the majority of the variation detected in the samples across two axes (Figure 4a). Axis 1 accounted for 34.6% of the variation, and Axis 2 accounted for 14.8% of variation. In Figure 4b, the same 2-dimensional plot was shown for the unweighted method which shows that in consideration of community membership, samples from the same type of land use system clustered together, although less distinctive (Axis 1 = 18.5%, Axis 2 = 14.1%).

The results from the Unifrac weighted and unweighted PCoA plots demonstrate the distinction that the bacterial communities under different land use management in both structure and composition. This is perhaps the most persuasive evidence that the three types of land use cultivate three distinctive bacterial communities. In the weighted PCoA plot (Figure 4a) the communities are clearly distinguished from one another quantitatively. There does appear to be some divergence within the samples from the cultivated system, as sample Cultivated 1, differs in its location on the y-axis. The divergence suggests that this sample exhibits different structure than the other two samples. This same phenomenon was evident when calculating the richness of the cultivated system, which explains the high error displayed in Figure 1a (as richness is considered a component of community structure). Regardless of its divergence, this community still seems distinct as clear separation is still detectable from the other land use types, primarily in the x-direction. The communities in the unweighted PCoA plot (constructed with respect to composition) show clear distinction as well (Figure 4b), as the samples from each of the land use systems cluster within the system. The pine forested and cultivated systems show the most dissimilarity with respect to structure and membership when considering the amount of space which separates them on the plot. Although the cultivated and pastured systems show the most similarity in physiochemical characteristics and richness/diversity values, they did show distinction in structure. Wardle [39] suggested that the difference in soil microbial communities of lightly disturbed (reduced/no till) and heavily disturbed (conventional tillage) systems is exemplified in the composition of the communities as seen in Figures 4a and 4b.

### Shared Observations

The Venn diagram presented in Figure S1 demonstrates the distribution of phylotypes at the 3% level of dissimilarity. The total number of phylotypes found in the entire agroecosystem is 7771. All three land use systems shared 2.2% of these phylotypes, while the most were shared between the pastured and cultivated systems (8.9%). The cultivated and forested systems share the least amount of phylotypes (2.5%), and the forested and pastured system share 4.5%.

Clustering results showed three discrete communities present under the agroecosystem. Though the grazed pasture and forested systems show the greatest dissimilarity in the data, it may be suggested that the grazed pasture and cultivated systems may show more similarity. The Venn diagram further establishes this point. Of the 11.2% of the phylotypes that the grazed pasture system shares, 8.9% is shared with the cultivated community, as compared to the 4.5% that it shares with the forested community. Even though these similarities are observed, the data also distinguishes the grazed pasture and cultivated systems in the Unifrac PCoA plots (Figures 4a & 4b). This distinction suggests that regardless of the phylotype sharing there are significant differences in the structure and membership of the communities. With the unique membership and structure elucidated by the three systems studied on a single soil type, the development of these communities with respect to management raises further questions as to the influence of environmental history and dispersal within the soil ecosystem.

In conclusion, components of this study further establish the distinction of soil bacterial communities with respect to differing land management strategies. As has been demonstrated in other studies using both 16S rRNA and PLFA techniques we notice distinction of forested soils with respect to community composition, structure, and phylogeny. The bacterial communities under in the cultivated and grazed pasture soils were similar in richness and composition, furthering the point that in a community with moderate disturbance, new individuals and groups could be introduced in a manner that promotes competition and diversity of the community, thus establishing a more stable community [47]. Though community richness and composition observed under the cultivated and grazed pasture regimes were not distinguishable, metrics assessing phylogenetic differences showed distinct communities under all three land use types. Further analysis sheds more light on the behavior of soil bacterial Orders and Families under differing land management strategies. As has been noted in other studies, phyla and class taxa behave according to predetermined guidelines regarding the chemical ecology of microbial communities, but at the lower levels of taxa a majority of groups depart from these generalizations. Further study will be needed to actually elucidate and validate the divergence in ecology of the descending taxa.

## Materials and Methods

### Sites and Sampling for Physical and Biochemical Analyses

The study site was located on the Sundown Ranch demonstration farm located at 32° 26′ latitude and −87° 27′ longitude on 17 hectares of land in Perry County, Alabama, USA. The soil series of the site was Kipling clay loam (Fine, smectitic, thermic Vertic Paleudalfs). For the past 10 years this land has been used as a demonstration farm for a consortium of Alabama Land Grant Universities.

A preliminary geostatistical study of soil biochemical characteristics provided the initial evidence that soil biochemical and biological factors spatially vary with respect to land use type on this site. The preliminary study allowed for the reasonable designation of three major sampling areas that coincided with land management considering the spatial variability of the soil surface. The sampling methodology of choice was a stratified random sampling design that utilized sampling areas that coincided with specific land use types. Effort was made to obtain as statistically useful data set as possible that was well distributed throughout the site, while achieving a cost effective sampling method. The three sampling areas were constructed of sizes which were reflective of the amount of area each covered of the farm and overlaid by sampling grids. Sample numbers were assigned to each vertex in the grids, and the actual sample locations were randomly selected from these grid points. The amount of samples collected were proportional to the land area each land use system covered (loblolly pine forest = 8 ha; grazed pasture = 5 ha; cultivated = 3 ha).

The grazed pastured area is where pasture poultry are grazed twice yearly, and goats graze moderately throughout the year. This has been the management practice for the past 5 years. The loblolly pine plantation, which accounted for approximately half of the total area of the site, was designated as the forested system. It is characterized by a pine plantation at about 15 years of growth, and is lightly grazed by a goat herd. The crop-growing area was designated as the cultivated system, was used annually as a mixed cropping system (corn, beans, tomatoes, squash, and okra). This area has been under differential cropping management during the 10 years with the last 5 years being plastic mulch, inorganic fertilizer, and no herbicide.

Sampling was conducted on 14 October 2008 following the summer growing season. Soil samples of approximately 120-g field-moist weight were systematically collected from the upper 15 cm of soil at 45 sampling points in the landscape using a soil auger. In between sampling, the auger was sterilized with ethanol. Samples were preserved on ice during transport to the laboratory. Upon arrival soils were shortly stored at field moist conditions at 4°C. Prior to biochemical and physical assays soils were air-dried for 48 h, plant residues were removed by hand and the soil was sieved using a 2 mm mesh and mixed thoroughly thereafter. Five grams of soil were separated and kept at 4°C for molecular analysis.

### Soil Enzyme Activity

Enzyme analysis was performed according to the methods of Tabatabai [48], with slight modification. The artificial substrate, *p*-nitrophenyl (1 mL, 0.05 M), and a pH buffer (pH values were 11 for Acid Phosphatase [*APA*], 6.5 for Acid Phosphatase [*ACP*], and 8 for Phosphodiesterase [*PD*]) were incubated in 25 mL glass flasks and capped at 37°C for 1 h with 1 g of soil. At the end of incubation, enzyme activity was stopped by addition of 4 mL of 0.5 M NaOH for phosphomonoesterases and 4 mL of 0.5 THAM-NaOH for phosphodiesterase followed by extraction with 1 mL of 0.5 M CaCl_2_. The mixture was then filtered (Whatman No. 2) and the extract analyzed using a Genesys 10 VIS spectrophotometer at (Thermo Fisher Scientific Inc., Waltham MA, USA) at 420 nm. Enzyme activity in filtrates was determined from a standard curve developed using p-nitrophenol standards. To account for non-enzymatic hydrolysis, values for controls were subtracted from sample readings. Toluene was not used in accordance with Bandick and Dick [49] and Elsgaard et al. [50], who showed that with incubation periods fewer than two hours, the absence of toluene was inconsequential to measured enzyme activity. All enzyme activities reported are expressed on a moisture-free basis.

### Soil pH and SOM

Soil pH was analyzed using a 1∶2 soil/water ratio according to those methods described in McLean [51]. The samples were then analyzed with an S500 pH Meter (following calibration at a pH of 4 and a pH of 10) [A. Daigger & Co., Vernon Hills, Illinois USA], measured to the nearest 0.01. For soil organic carbon (SOC) and total nitrogen (TN), air-dried soils were transferred to Auburn University Soil Testing Laboratory for analysis by the dry combustion method using an Elementar Vario Macro Combustion Analyzer (Elementar Americas, Inc., Mt. Laurel, NJ).

### Soil Textural Analysis

Soil textural analysis was determined using the Buyocos hydrometer method for 40 g duplicate sample and <2 mm diameter. To enhance dispersion of the soil particles chemically, 100 mL of 0.05 g mL–1 Sodium Hexametaphosphate (HMP) solution was added to each sample and shaken (for mechanical dispersion) for 12 h [52]. After shaking, the HMP solution and soil were transferred into a 1000 mL sedimentation cylinder. Deionized water was added to bring the total volume in the sedimentation cylinder to 1000 mL. To begin sedimentation process, the sedimentation cylinder containing the sample was agitated by shaking the cylinder back and forth for a minimum of 30 s. During the agitation, care was taken to ensure that particles were not stuck on the sedimentation cylinder. After agitation the cylinder was placed on the countertop, signifying time zero. Readings were then taken at elapsed times of 0.667, 3, 10, 30, 90, 120, and 720 min using a 152 H hydrometer (H-B Instrument Company, Collegeville, Pennsylvania, USA). Temperature of the suspension liquid was recorded simultaneously with hydrometer readings. To calibrate the hydrometer, a blank reading was taken in a solution containing 0.05 g mL–1 100 mL of Sodium HMP solution and 900 mL deionized water, but without soil.

### DNA Extraction, Amplification, and Sequencing

For molecular analysis of community DNA, nine soil samples were randomly selected from the soil samples within each of the three strata. DNA was extracted from these soil samples for each of the three strata and composited following extraction and validation (providing three representative samples per land use system). As previously demonstrated in other 16S rRNA studies [11], [31], [35], DNA was extracted from approximately 0.25 g of soil (oven dry basis of field-moist soil) using the Power Soil Extraction Kit (MO BIO Laboratories, Soloana Beach, California) according to the included protocol. Extracted DNA (2 µL) was quantified using Nanodrop ND-1000 spectrophotometer (Nanodrop Technologies, Wilmington DE), and run on 0.8% agarose gel with 0.5 M TBE buffer. The samples were then submitted to Research and Testing Laboratories (Lubbock, TX) for PCR optimization and pyrosequencing analysis. Bacterial tag-encoded FLX amplicon pyrosequencing PCR, massively parallel pyrosequencing and tag design were carried out according to procedure described previously by Dowd et al. [53], [54].

Samples were evaluated using Tag-encoded FLX amplicon pyrosequencing (bTEFAP), which has had prior description and utilization by Dowd et al. in characterizing bacterial populations in a variety of studies [28], [54], [55]. All DNA samples were diluted to 20 ng/µl. A 20 ng (1 µl) aliquot of each sample DNA was used for a 25 µl PCR reaction with 5 min denature at 95°C, 30 cycles of 94°C 30 sec –52°C 40 sec –70°C 40 sec with a final extension of 70°C for 5 minutes. The 16S universal Eubacterial primers 28 F (5′- GGC GVA CGG GTG AGT AA) and 530 R (5′-CCG CNG CNG CTG GCA CS) Amplicons were mixed in equal volumes and purified using Agencourt Ampure beads (Agencourt Bioscience Corporation, MA, USA). In preparation for FLX sequencing (Roche, Nutley, NJ), the DNA fragments size and concentration were measured by using DNA chips under a Bio-Rad Experion Automated Electrophoresis Station (Bio-Rad Laboratories, Hercules, CA) and a TBS-380 Fluorometer (Promega Corporation, Madison, WI). A 9.6×10^6^ sample of double-stranded DNA molecules/µl with an average size of 625 bp were combined with 9.6 million DNA capture beads, and then amplified by emulsion PCR. After bead recovery and bead enrichment, the bead-attached DNAs were denatured with NaOH, and sequencing primers were annealed. The 454 Titanium sequencing run was performed on a 70×75 GS PicoTiterPlate by using a Genome Sequencer FLX System (Roche, Nutley, NJ).

### Bioinformatics and Statistical Analysis

Quality trimmed sequences were provided with the sequencing services by Research and Testing Laboratories (Lubbock, TX) following the process described in Acosta-Martinez et al. [11]. As described in the aforementioned studies, each sequence was trimmed to utilize only high quality sequence information; tags were extracted from the FLX generated multi-FASTA file, while being parsed into individual sample specific files based upon the tag sequence. Tags which did not have 100% homology to the original sample tag designation were not considered. Sequences which were less than 250 bp after quality trimming were not considered. The B2C2 software [56], which is described and freely available from Research and Testing Laboratory (Lubbock, TX, USA), was used to deplete samples of definite chimeras. Further processing and OTU based analyses were then carried out using the MOTHUR v.1.19.4 [57] suite of algorithms for sequence processing and diversity analysis, including commands for identifying/consolidating unique sequences, filtering, multiple sequence alignment, generating distance matrices, and clustering of sequences into OTUs. The resulting clusters were assessed at 3% and 5% dissimilarity to provide the data needed for diversity analysis. Based upon the literature we can expect that 0% dissimilarity in sequences will provide dramatic overestimation of the species present in a sample, based upon rarefaction [10]. The resulting sequences were then evaluated using the classify.seqs algorithm (Bayesian method) in MOTHUR against a database derived from the Greengenes set using a bootstrap cutoff of 65%. The sequences contained within the curated 16S database were those considered of high quality based upon Greengenes [58] standards and which had complete taxonomic information within their annotations. Clusters at 3% and 5% were then utilized to generate rarefaction curves and the (diversity) indices ACE [59] and Chao1 [60] as well as unweighted and weighted UniFrac for Principle Coordinate Analysis (PCoA) plots and a venn diagram.

All statistical analysis was performed using the SPSS package (SPSS Inc, v 17.0, Chicago, Illinois). The generalized linear model (GLM) was used to assess the means of soil physical, chemical and microbial properties among the systems followed by a Tukey’s HSD test for pairwise comparisons. Relative abundance data is presented as percentages/proportions, but prior to subjection to GLM, they were transformed using the arcsine function for normal distribution prior to analysis. NCSS package (NCSS, 2007, v 7.1.2, Kaysville, Utah) was used for cluster analysis through which double dendrograms were generated through use of the Manhattan distance method with no scaling, and the unweighted pair technique.

Raw sequences were submitted to the NCBI Sequence Read Archive (SRA) and can be found under the accession number SRA007616.

## Supporting Information

Figure S1 **Venn diagram of shared OTUs.** A venn diagram of the phylotype richness among the three land use systems at 3% dissimilarity. The size of the spheres is not consistent with the amount of phylotypes present.
(TIF)
