# Supplementary figures and images for: Distinct Soil Bacterial Communities Revealed under a Diversely Managed Agroecosystem
Source: PLoS One. 2012 Jul 23;7(7):e40338. doi: 10.1371/journal.pone.0040338 (PMC3402512; doi:10.1371/journal.pone.0040338)

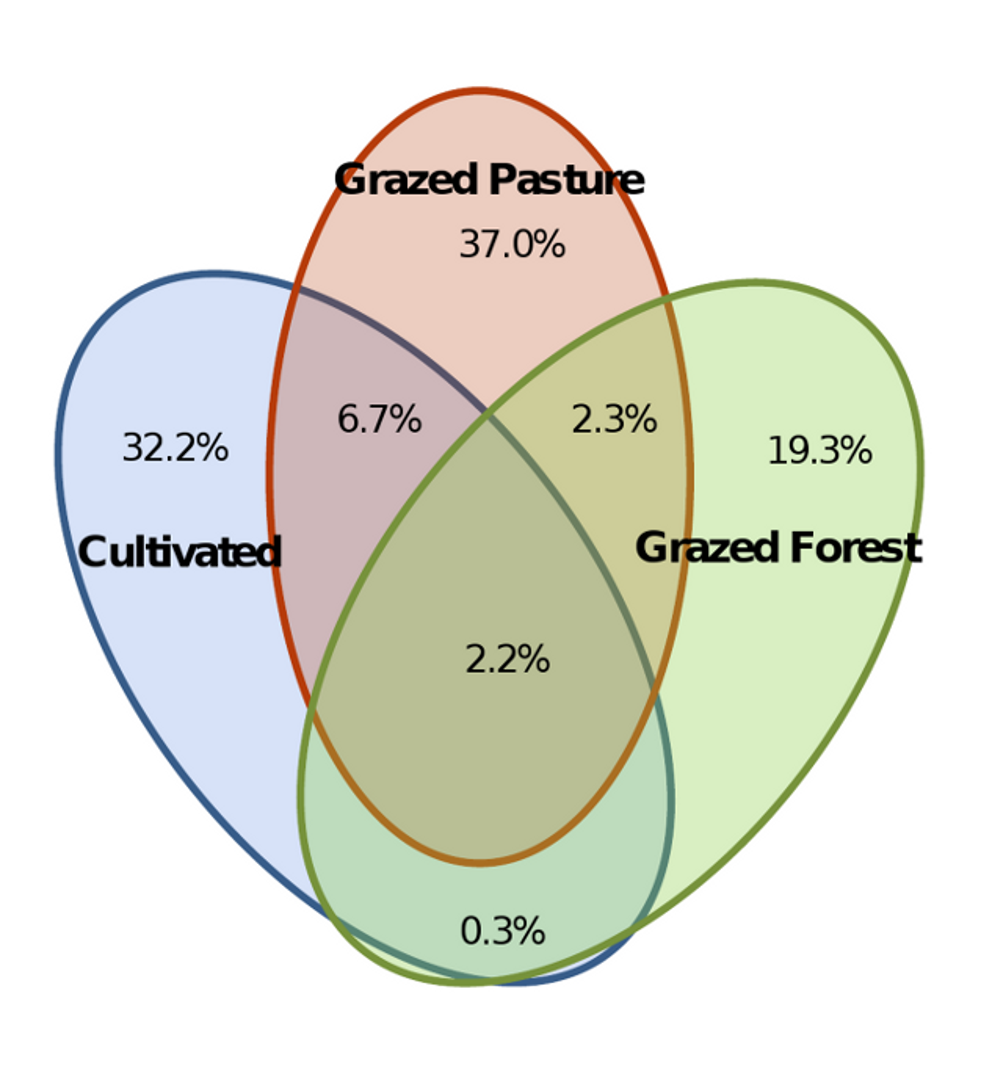

Supplement: Figure S1 — Venn diagram of shared OTUs. A venn diagram of the phylotype richness among the three land use systems at 3% dissimilarity. The size of the spheres is not consistent with the amount of phylotypes present. (TIF) [file pone.0040338.s001.tif]
